# Die Rolle des Internets als medizinische Informationsquelle für orthopädische Patienten

**DOI:** 10.1007/s00132-022-04238-5

**Published:** 2022-03-29

**Authors:** Stefan Hertling, Georg Matziolis, Isabel Graul

**Affiliations:** 1 Abteilung für Frauenheilkunde und Fortpflanzungsmedizin, Universitätsklinikum Jena, Am Klinikum 1, 07747 Jena, Deutschland; 2 Deutsches Zentrum für Orthopädie, Campus Eisenberg, Universitätsklinikum Jena, Eisenberg, Deutschland; 3 Klinik für Orthopädie und Unfallchirurgie, Universitätsklinikum Halle, Halle, Deutschland

**Keywords:** COVID-19, Gesundheitsbildung, Orthopädie, Umfrage, Web-Nutzung, COVID-19, Health education, Orthopedics, Survey, Web use

## Abstract

**Einführung:**

Der Vormarsch der digitalen Revolution im Medizin- und Gesundheitsbereich wird als „e-health“ bezeichnet. Das Internet dient als digitale Gesundheitsinformationsplattform, da eine medizinische Informationsquelle unverzichtbar ist. Ziel dieser Arbeit ist es, orthopädische Patienten auf ihr Informationsverhalten über ihre Erkrankungen zu untersuchen. Dabei werden die Rolle und Bedeutung des Internets als informative Gesundheitsanwendung für diese Patienten beleuchtet.

**Materialen und Methoden:**

Grundlage dieser prospektiven Querschnittsstudie war eine Patientenbefragung in Deutschland bei orthopädischen Patienten von Juli 2019 bis Juli 2020. Es wurden deskriptive Statistiken berechnet und Regressionsanalysen durchgeführt, um Zusammenhänge aufzuzeigen.

**Ergebnisse:**

Wir analysierten die Antworten von 1262 orthopädischen Patienten. Die meisten von ihnen nutzen das Internet als digitale Gesundheitsinformationsplattform. Ihre Internetkenntnisse bewerteten die Patienten unabhängig von Alter oder Bildungsstand als gut bis sehr gut. Die meisten Befragten gaben an, dass sie derzeit mindestens einmal pro Woche das Internet nutzen, um sich über ihre orthopädische Erkrankung zu informieren. Patienten berichteten, dass sich ihre Einstellung zum Internet als digitale medizinische Informationsquelle positiv verändert und dessen Nutzung in den letzten 12 Monaten zugenommen hat.

**Schlussfolgerung:**

Das Internet als informative digitale Gesundheitsanwendung in der Orthopädie wird intensiv genutzt und von den Patienten weitgehend akzeptiert. Während das Misstrauen gegenüber orthopädischen Gesundheitsinformationen aus dem Internet abgenommen hat, stieg aus Patientensicht das Vertrauen in digitale Gesundheitsinformationsplattformen. Das Internet wurde neben der ärztlichen Beratung als hilfreiche Gesundheitsinformationsplattform gesehen.

## Abstract (en)

**Introduction:**

The advance of the digital revolution in medicine and health is called “e-health”. The Internet serves as a digital health information platform and is indispensable as a source of medical information. The aim of this work was to examine the behavior of orthopedic patients with respect to finding information on their illnesses. The role and the importance of the Internet as an informative health application for these patients are highlighted.

**Materials and methods:**

The basis of this prospective cross-sectional study was a patient survey in Germany of orthopedic patients from July 2019 to July 2020. Descriptive statistics were calculated, and regression analyses were carried out to show coherent results.

**Results:**

We analyzed the responses of 1262 orthopedic patients. Most of them used the Internet as a digital health information platform. Patients rated their Internet skills as good to very good, regardless of age or educational level. Most respondents said that they currently use the Internet at least once a week to find out about their orthopedic illnesses. Patients reported a positive change in attitude towards the Internet as a digital source of medical information, and its use has increased in the past 12 months.

**Conclusion:**

The Internet as an informative digital health application in orthopedics is used intensively and is widely accepted by patients. While mistrust of orthopedic health information from the Internet has decreased, patient confidence in meaningful digital health information platforms has increased. The Internet is seen as a useful health information platform alongside medical advice.

## Einführung

Das Fortschreiten der digitalen Revolution im Medizin- und Gesundheitssektor wird als „e-health“ bezeichnet [1]. Dabei spielen verschiedene Begriffe eine entscheidende Rolle. Die Vernetzung der medizinischen Versorgung wird auf allen Ebenen mithilfe moderner Informations- und Kommunikationstechnologien [2] bearbeitet. Digitalisierung bezeichnet nicht nur die Bewahrung von Informationen in elektronischen Medien, sondern auch die Vernetzung von Einzeldaten [3]. Hierbei werden analoge Daten durch digitale, elektronische Daten mit dem Ziel der Automatisierung ersetzt [4]. Dabei spielt das World Wide Web (WWW) eine wichtige Rolle: Das Internet ist die zweitwichtigste Informationsquelle für deutsche Bürgerinnen und Bürger und hat sich damit zu einem bedeutenden Informationsmedium entwickelt [5]. Alltägliche Informationen werden über das Internet eingeholt. Dazu werden regelmäßig verschiedene Suchmaschinen im Internet genutzt. Im Gesundheitswesen werden Informationen über Symptome, Erkrankungen und Diagnosen gewonnen. Patienten können diese Informationen über verschiedene Informationsplattformen erhalten und medizinische Fragen beantworten. „Dr. Google“ gilt als Pseudonym [6]. Das Internet dient als digitale Gesundheitsinformationsplattform und ist als medizinische Informationsquelle aus Patientensicht unverzichtbar [6].

Verschiedene Determinanten beeinflussen dabei das Informationsverhalten im WWW: Durch die Veränderung des gesellschaftlichen Rollenmodells des Arztes nehmen die Patienten eine aktivere Rolle in der Arzt-Patienten-Beziehung ein [7]. Dies führt zu einem Rollenwechsel aus Sicht des Patienten: von dem unwissenden hin zum unabhängigen, gut informierten, sowie selbst- und mitbestimmenden Patienten [8]. Aufgrund des digitalen Wandels in der Medizin steigen damit auch die Erwartungen an die Versorgungsqualität und der Erfolgsdruck in der Medizin. Es ist nicht ungewöhnlich, dass Patienten sich vor dem Arztbesuch über ihr eigenes Krankheitsbild und ihre Symptome informieren [9]. Es bleibt dem Patienten überlassen, in welchem Umfang er digitale Gesundheitsanwendungen als medizinische Informationsunterstützung nutzen möchte [10]. Die Einsatzmöglichkeiten im medizinischen Bereich sind praktisch unbegrenzt und als Informationsquelle bei Patienten sehr beliebt [11]. Seit der COVID-19-Pandemie sind digitale Informationen aus dem Internet in allen Lebensbereichen unverzichtbar. Diese Veränderung zeigt sich auch im Gesundheitswesen [12].

Ziel dieser Arbeit ist es, Patienten, bei denen eine elektive orthopädische ambulante Behandlung nötig war, nach ihrem Informationsverhalten zu ihren muskuloskelettalen Symptomen zu befragen. Die Rolle und Bedeutung des Internets als digitale Gesundheitsinformationsplattform für diese Patienten soll herausgearbeitet werden. Neben Fragen zur generellen Nutzung digitaler Medien und zur Wahrnehmung von „e-health“ wird untersucht, woher die befragten Patienten Informationen über ihre orthopädischen Erkrankungen erhalten und wie sie die Vertrauenswürdigkeit der einzelnen Informationsquellen einschätzen. In diesem Zusammenhang soll die Rolle digitaler Gesundheitsinformationsplattformen, insbesondere des Internets als „e-health“-Anwendung, aus der Perspektive der befragten Patienten betrachtet werden. Mögliche Einflussgrößen (Alter, Bildungsstand, ausgeübte Berufstätigkeit) auf das Informationsverhalten der Patienten sowie deren Einsatz und die Einstellung zu „e-health“ sollen bewertet werden.

## Material und Methoden

Es wurde eine Befragung zur Nutzungssituation des Internets als digitale Gesundheitsinformationsplattform unter orthopädischen Patienten durchgeführt. Die zuständige Ethikkommission der Universitätsklinik Jena wurde informiert und hatte keine Einwände gegen die Studie (Reg.-Nr.:2019-1456-Bef). Die papierbasierte Befragung wurde von Mitgliedern der Arbeitsgruppe Digitalisierung der Deutschen Gesellschaft für Orthopädie und Unfallchirurgie (DGOU) erstellt. Um die identifizierten Interessengebiete zu untersuchen, hat ein Expertengremium nach individueller Literaturrecherche in Analogie zur EULAR Recommendation Task Force Standard Operations Procedures in zwei separaten Online-Meetings einen Fragebogen entwickelt [13]. Vier Bereiche wurden untersucht:

- epidemiologische Daten der Befragten
- grundlegende Nutzung digitaler Anwendungen
- Nutzung der digitalen informativen Gesundheitsapplikationen
- Internet als digitale informative Gesundheitsanwendung

Ziel der Befragung war es, die Interviewdauer auf maximal 15 min zu verkürzen, um die Abbruchquote so gering wie möglich zu halten und die Befragten zu motivieren, möglichst viele Fragen zu beantworten [14]. Der Fragebogen wurde als Papierfragebogen an die Patienten verteilt. Dieser wurde den Patienten vor ihrer elektiven ambulanten Behandlung ausgegeben. Die Teilnehmer wurden darauf hingewiesen, dass ihre Daten streng vertraulich und anonym bleiben würden. Alle Teilnehmer gaben ihr Einverständnis. Ausschlusskriterien für die Teilnahme gab es nicht. Nur vollständig ausgefüllte Fragebögen wurden in die anschließende Analyse einbezogen. Die Papierbefragung wurde von Juli 2019 bis zum Juli 2020 durchgeführt. Die Studie wurde im Einklang mit den geltenden Datenschutzbestimmungen und der Helsinki-Erklärung durchgeführt.

### Auswertung

Die Auswertung der Ergebnisse erfolgte mit Microsoft Office Excel 2021 (Microsoft, Redmont, WA, USA) und dem Statistical Package for the Social Sciences, SPSS (Version 21.0, SPSS Inc., Chicago, IL, USA). Deskriptive Statistiken umfassten Mengen, Prozentsätze, Medianwerte und Bereiche für ordinale Variablen. Für die Korrelation der Variablen mit Alter oder Schulabschluss wurde der Spearman-Test oder Kruskal-Wallis-Test verwendet. Die *p*-Werte wurden mit dem Mann-Whitney-U-Test berechnet. Ein *p*-Wert von weniger als 0,05 wurde als signifikant angesehen.

### Inhalt des Studienfragebogens

Ein Papierfragebogen mit 22 Fragen wurde eigens für diese Studie konzipiert. Mitglieder der Arbeitsgemeinschaft Digitalisierung der DGOU wurden für den Validierungsprozess eingeladen, um Feedback zu Fragenformat, Vollständigkeit und Verständlichkeit zu geben [15]. Die Umfrage wurde an 15 Patienten getestet, um die Notwendigkeit von Veränderungen von Wortlaut und Format zu beurteilen, und zu prüfen, ob die vordefinierten Antwortoptionen passend waren. Überarbeitungen wurden vorgenommen und der Fragebogen wurde modifiziert. Er bestand aus binominalen Fragen, Fragen in kategorischen Likert-Skalen und offenen Fragen. Der Titel trug den Titel „Internet als informative digitale Gesundheitsanwendung“.

## Ergebnisse

### Überblick

Von September 2019 bis September 2020 erfolgte eine papierbasierte Umfrage zu Akzeptanz, Nutzung und Barrieren des Internets als digitale Gesundheitsinformationsplattform unter Patienten mit muskuloskelettalen Erkrankungen in Deutschland.

Von den 5270 ausgegebenen Patientenfragebögen wurden 1659 (31 %) zurückgegeben. Von den 1659 wurden 397 (24 %) aus der Analyse ausgeschlossen, da nicht alle Fragen beantwortet wurden. Die endgültige Antwortrate der Patienten betrug 23 % (1262/5270). Im Zeitraum von September 2019 bis September 2020 haben wir in unserer Abteilung mehr als 41.000 Patienten aufgrund einer muskuloskelettalen Erkrankung ambulant behandelt. An der Studie nahmen 1262/41.000 (3,1 %) Patienten teil.

### Demografische Daten der Befragten

**Figure 1:**
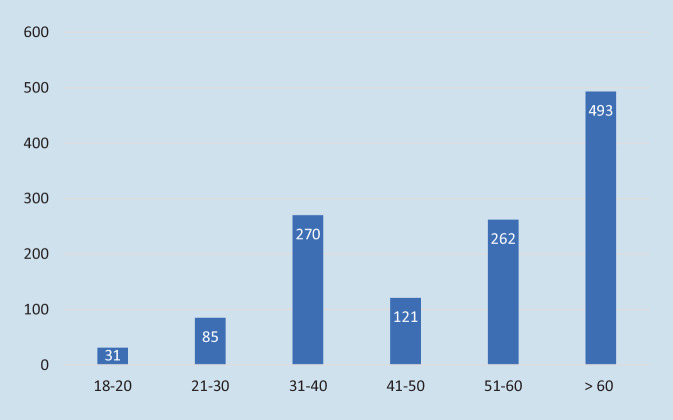

**Figure 2:**
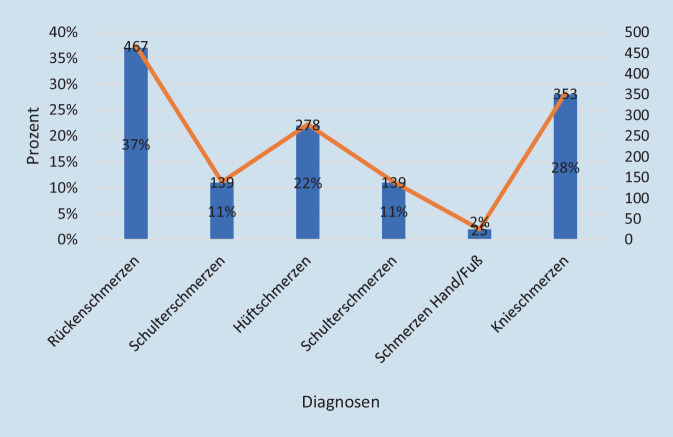

1262 Patienten nahmen an der Papierumfrage teil. Zwei Altersgipfel sind vertreten: 21,4 % (*n* = 270) 31–40 Jahre und 39,1 % (*n* = 493) ab 60 Jahre (Abb. 1). Das Durchschnittsalter der teilnehmenden Patienten betrug 52,4 Jahre ± 16,8 und die Mehrheit war männlich (*n* = 717, 56,8 %). Rückenschmerzen (*n* = 467, 37 %) und Knieschmerzen (*n* = 353, 28 %) sind die häufigsten Erkrankungen (s. Abb. 2).

Der erreichte Schulabschluss wurde abgefragt und die Einteilung nach Abitur, Realschulabschluss und Hauptschulabschluss sowie „kein Zeugnis“ angewendet. Befragt nach dem Bildungsniveau gaben 707 (56 %) der Befragten an, einen Realschulabschluss zu haben. 35 % (*n* = 442) von ihnen hatten einen Hauptschulabschluss. 4 % (*n* = 50) der Patienten hatten keinen Schulabschluss. 5 % (*n* = 63) der Befragten verfügten über ein Abitur. 5 % (*n* = 63) der Befragten verfügten über einen Hochschulabschluss.

### Grundlegende Nutzung digitaler Anwendungen

88,1 % (*n* = 1112) der Patienten gaben an, ein internetfähiges Gerät zu besitzen. Davon haben über 75 % (*n* = 834) ein Smartphone, um online zu gehen. 176 Patienten (15,8 %) gehen lieber mit einem Tablet online. Fast 10 % der Befragten (*n* = 102) bevorzugen einen PC oder Laptop oder andere internetfähige Geräte. Fast 80 % (*n* = 1009) der Teilnehmer fühlen sich in der Lage, internetfähige Geräte zu nutzen. Nur 5 % (*n* = 63) gaben an, sich nicht in der Lage zu fühlen, internetfähige Geräte zu nutzen. Zudem geben fast 67 % (*n* = 846) an, dass sich ihre Einstellung zu internetfähigen Geräten in den letzten 3 Jahren positiv verändert hat. Auf die Frage, wie oft die Befragten ihre internetfähigen Geräte nutzten, gaben fast 80 % (*n* = 885) an, dies mehrmals täglich zu tun. Nur 6 % (*n* = 67) gaben an, weniger als einmal die Woche internetfähige Geräte zu nutzen (Tab. 1).

**Table 1:** 

Merkmale	Patienten *n* = 1262 (100 %)
*Haben Sie ein internetfähiges Gerät? (Smartphone, Tablet, Computer …)*	
Ja	1112 (88,1)
Nein	150 (11,9)
*Mit welchem Gerät gehen Sie bevorzugt online?*	
Smartphone	834 (75)
Tablet	176 (15,8)
PC/Laptop	76 (6,9)
Andere	26 (2,3)
*Ich fühle mich in der Lage internetfähige Geräte zu benutzen.*	
Lehne ich vollstens ab	15 (1,2)
Lehne ich ab	48 (3,8)
Neutral	189 (15,1)
Stimme ich zu	731 (57,9)
Stimme ich voll und ganz zu	278 (22)
*Hat sich in den letzten drei Jahren Ihre Einstellung zu internetfähigen Geräten verändert?*	
Positiv verändert	846 (67,1)
Negativ verändert	261 (20,7)
Gleichbleibend	155 (12,2)
*Wie oft benutzen Sie ihr internetfähiges Gerät?*	
Mehrmals am Tag	885 (79,6)
Einmal täglich	88 (7,9)
Einmal die Woche	72 (6,5)
Nie	67 (6,0)

### Digitale Gesundheitsanwendungen: Wissen und Anwendung

84 % (*n* = 1060) der Patienten können digitale Gesundheitsanwendungen nutzen. Knapp 70 % (*n* = 877) geben an, dass die Nutzung digitaler Gesundheitsanwendungen einen positiven Einfluss auf ihre Krankheitsbehandlung haben kann, während knapp 11 % (*n* = 139) dem nicht zustimmen. Die Einstellung zu digitalen Gesundheitsanwendungen hat sich bei 52 % der Patienten (*n* = 656) in den letzten 3 Jahren positiv verändert. Über 68 % der Patienten (*n* = 858) berichten, dass sie regelmäßig digitale Gesundheitsanwendungen nutzen (Tab. 2). Je höher das Alter der Patienten, desto geringer ist deren Nutzung insgesamt (*p* < 0,001) und desto geringer ist ihr Vertrauen in die Nutzung digitaler Gesundheitsanwendungen (*p* < 0,001). Es wurde kein signifikanter Unterschied beim Schulabschluss festgestellt (*p* = 0,345).

**Table 2:** 

Merkmale	Patienten *n* = 1262 (100 %)
*Ich fühle mich in der Lage digitale Gesundheitsanwendungen zu nutzen?*	
Lehne ich voll und ganz ab	14 (1,1)
Verneine ich	77 (6,1)
Neutral	111 (8,8)
Stimme ich zu	1060 (84)
*Ich glaube, dass die Nutzung digitaler Gesundheitsanwendungen einen positiven Einfluss auf meine Krankheitsbehandlung haben kann.*	
Lehne ich voll und ganz ab	57 (4,5)
Lehne ich ab	82 (6,5)
Neutral	246 (19,5)
Stimme ich zu	694 (55)
Stimme ich voll und ganz zu	183 (14,5)
*Hat sich Ihre Einstellung zu digitalen Gesundheitsanwendungen in den letzten drei Jahren verändert?*	
Es hat sich positiv verändert	656 (52,1)
Es hat sich negativ verändert	197 (15,6)
Es hat sich nicht verändert	409 (32,4)

### Internet als digitale Gesundheitsinformationsplattform

66 % der Patienten (*n* = 832) nutzen das Internet als Quelle für orthopädische Gesundheitsprobleme. Über 90 %von ihnen (*n* = 752) nutzen dafür regelmäßig das Internet. Die Patienten wurden gefragt, wie vertrauenswürdig sie Gesundheitsinformationen aus dem Internet auf einer Skala von 1–6 einstufen, wobei die 6 für sehr vertrauenswürdig und 1 für nicht glaubwürdig steht. Die Vertrauenswürdigkeit wurde von den Patienten mit durchschnittlich 4,8 bewertet. Es gab keine signifikanten Unterschiede in den Altersgruppen (*p* = 0,195) oder den unterschiedlichen Schulabschlüssen (*p* = 0,287). Die Patienten wurden gefragt, wie hilfreich sie die Gesundheitsinformationen aus dem Internet auf einer Skala von 1–6 einstufen. Wobei die 1 für wenig hilfreich und die 6 für sehr hilfreich steht. Die Patienten bewerteten die Hilfe im Durchschnitt mit 4,9. Signifikante Unterschiede gab es in den Altersgruppen (*p* < 0,001) mit zunehmendem Nutzen bei jungen Patienten (< 41 Jahre). Ausgehend von der Frage, ob der Schulabschluss die Wertwahrnehmung von Internetgesundheitsinformationen verändert, zeigt sich kein signifikanter Unterschied in der Nützlichkeit von Online-Informationen in den Gruppen mit unterschiedlichen Schulabschlüssen (*p* = 0,207). Über 53 % der Patienten (*n* = 669) gaben an, dass das Internet in den letzten 3 Jahren für sie zu einer wichtigen Quelle für Gesundheitsinformationen geworden sei. Bei muskuloskelettalen Beschwerden nutzen zwei Drittel (565/832) das Internet als Gesundheitsinformationsquelle, bevor sie zum Facharzt gehen.

### Konformität von orthopädischen Gesundheitsinformationen aus dem Internet mit Fachärzten

Befragt nach der Übereinstimmung der Online gefundenen Informationen zu orthopädischen Gesundheitsthemen und der Fachärzte (Orthopäden) ergab sich für alle Altersgruppen unter 60 Jahren eine Übereinstimmung von rund 74 %. Hingegen betrug die Übereinstimmung bei den über 60-Jährigen knapp 34 % und erreichte keine Signifikanz (*p* = 0,192). Vergleicht man die Angaben der Patienten zwischen den Online-Informationen und den orthopädischen Fachärzten hinsichtlich der verschiedenen Schulabschlüsse, liegt die höchste Übereinstimmung bei Patienten mit Abitur (85 %; 54/63), gefolgt von Patienten mit Realschulabschluss (78 %; 551/707) und Hauptschulabschluss (72 %; 318/442) sowie Patienten ohne Schulabschluss (65 %; 33/50) vor. Signifikante Unterschiede (*p* = 0,093) zeigen sich in diesem Zusammenhang nicht auf.

## Diskussion

### Hauptergebnisse

Ziel dieser Studie ist es, die Verfügbarkeit und den subjektiven Patientenwert der Online-Informationen zu orthopädischen Gesundheitsthemen sowie deren Konsistenz mit den Angaben des Facharztes zu bewerten. Darüber hinaus wurde untersucht, wie vertrauenswürdig die aus dem Internet gewonnenen Gesundheitsinformationen zu muskuloskelettalen Symptomen von den Patienten eingeschätzt werden und welche interindividuellen Determinanten dies beeinflussen können. Das Internet wurde von orthopädischen Patienten als hilfreiche Gesundheitsinformationsplattform gesehen. Es konnte gezeigt werden, dass bei 88,1 % der Patienten internetfähige Geräte vorhanden waren, jedoch mit zunehmendem Alter und abnehmender Schulbildung diese seltener Anwendung finden. Das anhaltende Wachstum des Internets hat Patienten einen einfachen Zugang zu medizinischen Informationen ermöglicht, unabhängig von Alter oder Schulabschluss. Bei der Informationssuche von Patienten mit orthopädischen Symptomen spielt das Internet eine wesentliche Rolle.

Eine Umfrage der Europäischen Kommission aus dem Jahr 2014 ergab, dass das Internet als Suchmedium für Informationen noch wichtiger ist: Hier haben sich 57 % der Deutschen in den letzten 12 Monaten im Internet über Gesundheitsthemen informiert. Damit liegt Deutschland leicht unter dem europäischen Durchschnitt von 59 % [16]. In dieser Studie gaben über zwei Drittel der Patienten an, das Internet als Informationsquelle zu nutzen. In den letzten Jahren hat die Bedeutung des Internets aus Patientensicht stetig zugenommen. Sie hat somit direkte Auswirkungen auf das Gesundheitssystem [17].

Es gibt viele Gründe für die Zunahme der Internetnutzung in Gesundheitsfragen. Ein Grund hierfür kann ein Mangel an Alternativen für die Patienten sein. Andere Informationsquellen zu Gesundheitsthemen, wie Bücher, stellen eine Barriere dar und sind aus Patientensicht weniger zugänglich [18]. Für die meisten Patienten sind Informationen aus Fachquellen aufgrund der verwendeten Fachterminologie oft irreführend oder unverständlich. In einer Studie von Ayantunde et al. gaben 70 % der Patienten an, Internetsuchmaschinen als erste Anlaufstelle für gesundheitliche Probleme zu nutzen [20]. Häufig erhalten Patienten auch medizinische Informationen über soziale Medien. Aufgrund dieser schwierigen Zugänglichkeit konnten sich seriöse Websites für die medizinische Informationseinholung aus Patientensicht noch nicht etablieren [19]. Neben Gesundheitssuchmaschinen und Lexika zur gezielten Recherche stellen auch private Unternehmen, Krankenhäuser und Arztpraxen ihre Informationen auf eigenen Homepages zur Verfügung [21].

Das Internet ist neben Haus- und Fachärzten, Zeitschriften und dem Fernsehen zu einer der wichtigsten Informationsquellen zu Gesundheitsthemen aus Patientensicht geworden [22]. Dabei spielt das Alter der Befragten eine wichtige Rolle. Diese Studie zeigte, dass das Internet für Patienten jeden Alters und vor allem für die Altersgruppe unter 60 Jahren eine wichtige Informationsquelle für orthopädische Gesundheitsfragen darstellt. Dieser Punkt wird in der Literatur kontrovers diskutiert. In einer Studie von Baumann et al. zeigte sich, dass die Wahrscheinlichkeit, Gesundheitsinformationen über das Internet zu erhalten, umso geringer war, je älter die Patienten waren [28]. Andere Studien zeigen jedoch, dass ältere Menschen zunehmend das Internet als Gesundheitsinformationsmedium nutzen.

In den USA ist ein Anstieg der älteren Nutzer von Gesundheitsinformationen von 24,8 % im Jahr 2009 auf 43,9 % im Jahr 2018 zu verzeichnen [23]. In der Studie von Hung et al. wurden neben dem Alter weitere Einflussfaktoren gefunden. Patienten mit höherer Bildung, höherem Einkommen oder hohem sozioökonomischem Status nutzen das Internet zunehmend als Informationsquelle für die Gesundheit [23]. In der vorliegenden Studie zeigte der Schulabschluss keinen signifikanten Einfluss auf den Abruf von Gesundheitsinformationen aus dem Internet bei den Patienten.

### Auswirkungen im klinischen Alltag

Viele Patienten informieren sich heute vor dem Arztbesuch [24]. In unserer Studie suchten über zwei Drittel der Patienten im Internet nach Gesundheitsinformationen zu ihren muskuloskelettalen Symptomen, bevor sie einen Arzt aufsuchten. Eine Information vor einem Arzt- oder Klinikbesuch kann die Arzt-Patienten-Beziehung beeinflussen. Nach wie vor übernehmen Patienten nicht mehr nur die Rolle des Laien, sondern haben durch die Vorabinformationen mehr Möglichkeiten, sich in das Gespräch einzubringen [25]. Patienten fühlten sich auch nach der Informationssuche in der Lage, kompetent an Entscheidungen mitzuwirken [26]. Patienten wird damit eine gewisse Emanzipation garantiert. Sie vertrauen dem Rat von Ärzten weniger blind und können Entscheidungen in Frage stellen.

Mittlerweile informiert sich fast ein Drittel der Patienten vor dem Arztbesuch über ihren Gesundheitszustand [27]. Eine weitere Studie zeigt eine noch höhere Zahl: 89 % der Befragten gaben an, dass sie sich in den letzten 12 Monaten über Fragen zu Gesundheitsthemen im Internet informiert haben. Ärzte befassen sich daher primär mit bereits informierten Patienten [28]. In dieser Studie suchten über zwei Drittel der Patienten im Internet nach Gesundheitsinformationen zu ihren muskuloskelettalen Symptomen, bevor sie einen Arzt aufsuchten. Dabei spielt die Unzufriedenheit von Patienten nach Arztkontakt eine große Rolle: Laut Baumann und Czerwinski suchen unzufriedene Patienten eher nach Gesundheitsinformationen im Internet [28]. Ein Grund kann die Verständnisbarriere zwischen Patienten und Arzt sein. Trotz vorab informierter Patienten fühlen diese sich auch nach einem Arztbesuch ungenügend oder schlecht informiert [29]. Dies könnte an der mangelnden Übereinstimmung von Gesundheitsinformationen aus dem Internet mit Spezialisten liegen.

Auf die Frage nach der Übereinstimmung zwischen den online gefundenen Informationen zum orthopädischen Gesundheitsthema und den Aussagen des Facharztes für Orthopädie, lag für alle Altersgruppen unter 60 Jahren eine Zustimmung von rund 64 % vor. Bei den über 60-Jährigen waren es hingegen nur 28 %. Bei den verschiedenen Schulabschlüssen war die Übereinstimmung bei Patienten mit Abitur höher als bei Patienten mit niedrigerem Schulabschluss. Diese Ergebnisse der Studie zeigen einen Zusammenhang zwischen den Faktoren Alter und Schulabschluss in Bezug auf die Konformität orthopädischer Gesundheitsinformationen aus dem Internet, verglichen mit den Aussagen von Fachärzten für Orthopädie. Dabei lassen sich keine signifikanten statistischen Ergebnisse darstellen. Zudem verändert sich das Rollenverhältnis zwischen Arzt und Patient: Der Patient kann sich im Vorfeld medizinisch behandeln lassen, indem er das Internet in Gesundheitsfragen nutzt oder sich über das Internet informiert und er kann sich gezielt für einen speziellen Facharzt entscheiden. Der Patient wird Koordinator und Verwalter seiner eigenen Untersuchungsergebnisse und Krankenakte. Daraus ergibt sich aus Sicht des Patienten ein Rollenwechsel: vom Unwissenden hin zum gut informierten, selbstbestimmten und stärker mitbestimmenden Patienten [30].

Auch die Kommunikationsfähigkeit des Facharztes spielt bei der subjektiven Einschätzung des Patienten eine große Rolle. Patientenorientierte Kommunikation spielt in der medizinischen Ausbildung oft nur eine untergeordnete Rolle, was auch zur Diskrepanz zwischen den Informationen beitragen kann [31]. Um diesen Sachverhalt abschließend beurteilen zu können, fehlte eine Befragung, wie vertrauenswürdig und hilfreich die Angaben der Spezialisten waren. Die Patienten in dieser Studie wurden gefragt, wie vertrauenswürdig sie Gesundheitsinformationen aus dem Internet einschätzen. Die Vertrauenswürdigkeit wurde von den Patienten mit durchschnittlich 4,6/6,0 bewertet. Es gab keine signifikanten Unterschiede in den Altersgruppen oder den unterschiedlichen Schulabschlüssen. Im Vergleich zu anderen Studien sind die Ergebnisse ähnlich. Obwohl die leichter zugänglichen Online-Informationen kritisiert wurden, die oft als verzerrt, unvollständig oder ungenau angesehen werden, vertrauen Patienten diesen Informationen und finden sie hilfreich [32]. Die Frage, ob Gesundheitsinformationen aus dem Internet als hilfreich angesehen werden, beantworteten die Patienten unserer Studie mit durchschnittlich 4,9 von 6 Punkten. Vor allem Patienten unter 60 Jahren bewerten die Informationen besser als Patienten über 60 Jahre. Dieser altersbedingte Unterschied könnte in den nächsten Jahren verschwinden, da jüngere Patienten, die mit dem Internet aufwachsen, die zukünftige ältere Bevölkerung werden und auf das Internet angewiesen sind [33]. In diesem Zusammenhang ist es zudem unklar, ob und wie die subjektive Wahrnehmung der Patienten bzgl. der Qualitätseinschätzung der online verfügbaren Informationen beeinflusst und ob Patienten ohne medizinische Ausbildung Fehlinformationen herausfiltern können [34].

### Einfluss von COVID-19

Die COVID-19-Pandemie hat positive Auswirkungen auf die Nutzung von „e-health“. Durch die COVID-19-Pandemie wird das Internet von mehr Menschen und häufiger genutzt. Ereignisse oder Prozesse finden vermehrt im digitalen Raum statt. Patienten nutzen seit der COVID-19-Pandemie häufiger digitale Gesundheitsanwendungen [35]. Diese Entwicklung zeigte sich auch in den Daten dieser Studie. Die befragten orthopädischen Patienten nutzen häufiger digitale Gesundheitsanwendungen. Dabei spielt das Internet für die orthopädischen Patienten eine wichtige Rolle in Gesundheitsfragen, sodass über zwei Drittel von ihnen das Internet als erste Anlaufstelle bei muskuloskelettalen Symptomen nutzen, bevor sie einen Facharzt aufsuchen.

Vergleichbare Studienergebnisse zu dieser Frage stehen noch aus, aber aus Sicht der Fachärzte spielen digitale Gesundheitsanwendungen für sie seit der COVID-19-Pandemie eine größere Rolle als vor der Pandemie. Die meisten Befragten finden den Einsatz digitaler Gesundheitsanwendungen für die Versorgung von Patienten hilfreich [36].

### Einschränkungen

Die Stärken der Studie sind die hohe Fallzahl, die konkrete Untersuchung des Internetverhaltens von orthopädischen Patienten und der Differenz bzw. Kohärenz der Informationen aus dem Internet mit denen des Arztes. Die Einschränkungen der Studie bestanden darin, dass eine objektive Bewertung der Informationen im Internet nicht möglich war. Zudem beantworteten nur orthopädische Patienten den Fragebogen. Daher kann der Effekt der Untersuchung des Einflusses von Internetinformationen nicht auf nichtelektive Pathologien übertragen werden. Bei der Patientenauswahl könnte ein Selektionsbias aufgetreten sein, da Patienten ohne Interneterfahrung eher nicht an der Studie teilnahmen.

## Zusammenfassung

Das Internet spielt eine immer wichtigere Rolle bei der Suche nach Gesundheitsinformationen aus Patientensicht. Das Internet als digitale Gesundheitsinformationsplattform bei auftretenden muskuloskelettalen Beschwerden wird intensiv genutzt und von den Patienten weitgehend akzeptiert. Das Misstrauen gegenüber orthopädischen Gesundheitsinformationen aus dem Internet hat abgenommen. In den letzten Monaten und Jahren hat die Bedeutung des Internets aus Patientensicht stetig zugenommen. Es hat somit einen direkten multidirektionalen Einfluss auf das Gesundheitswesen. Das Internet kann als digitale Gesundheitsinformationsplattform für Patienten mit muskuloskelettalen Beschwerden dienen. Verschiedene Einflussfaktoren können das Informationsverhalten der Patienten beeinflussen. Weitere Forschungen in diesem Bereich stehen noch aus und sollten durch groß angelegte, multizentrische Studien ergänzt werden.

## Abkürzungen

DGOU: Deutsche Gesellschaft für Orthopädie und Unfallchirurgie
e-health: electronic health
WWWW: World Wide Web
